# A Video Target Tracking and Correction Model with Blockchain and Robust Feature Location

**DOI:** 10.3390/s23052408

**Published:** 2023-02-22

**Authors:** Yiru Jiang, Dezhi Han, Mingming Cui, Yuan Fan, Yachao Zhou

**Affiliations:** 1 College of Information Engineering, Shanghai Maritime University, Shanghai 201306, China; 2 Hangzhou Anheng Information Technology Co., Ltd., Hangzhou 310051, China

**Keywords:** decentralized surveillance, secure tracking of objects, privacy-preserving target tracking, target correction, trajectory optimization

## Abstract

In this paper, a cutting-edge video target tracking system is proposed, combining feature location and blockchain technology. The location method makes full use of feature registration and received trajectory correction signals to achieve high accuracy in tracking targets. The system leverages the power of blockchain technology to address the challenge of insufficient accuracy in tracking occluded targets, by organizing the video target tracking tasks in a secure and decentralized manner. To further enhance the accuracy of small target tracking, the system uses adaptive clustering to guide the target location process across different nodes. In addition, the paper also presents an unmentioned trajectory optimization post-processing approach, which is based on result stabilization, effectively reducing inter-frame jitter. This post-processing step plays a crucial role in maintaining a smooth and stable track of the target, even in challenging scenarios such as fast movements or significant occlusions. Experimental results on CarChase2 (TLP) and basketball stand advertisements (BSA) datasets show that the proposed feature location method is better than the existing methods, achieving a recall of 51% (27.96+) and a precision of 66.5% (40.04+) in the CarChase2 dataset and recall of 85.52 (11.75+)% and precision of 47.48 (39.2+)% in the BSA dataset. Moreover, the proposed video target tracking and correction model performs better than the existing tracking model, showing a recall of 97.1% and a precision of 92.6% in the CarChase2 dataset and an average recall of 75.9% and mAP of 82.87% in the BSA dataset, respectively. The proposed system presents a comprehensive solution for video target tracking, offering high accuracy, robustness, and stability. The combination of robust feature location, blockchain technology, and trajectory optimization post-processing makes it a promising approach for a wide range of video analytics applications, such as surveillance, autonomous driving, and sports analysis.

## 1. Introduction

The field of target tracking technology has experienced significant advancements in recent years, providing a robust foundation for visual processing systems. A notable example of this was demonstrated during the opening ceremony of the 2022 Winter Olympic Games in Beijing, where 3D athlete tracking technology was used to create a mesmerizing snowflake effect, showcasing the remarkable improvement in this technology. The technology behind target tracking involves the integration of advanced algorithms, machine learning, and computer vision to create sophisticated systems capable of accurately detecting and tracking targets in real-time.

The research on target location has progressed significantly since the introduction of manual feature recognition algorithms in 1991. In particular, the integration of Convolutional Neural Network (CNN) features in tracking benchmark models has resulted in outstanding performance on target tracking datasets. The efficiency of visual target tracking on the OTB-2015 dataset has increased from 56.8% area under the curve (AUC) of success in 2017 to 71.9% in 2022.

Despite these advancements, there remain certain limitations to current target tracking algorithms, including the potential for cumulative inaccuracies due to errors in the tracking process. To address these limitations, various methods have been proposed. Guan et al. [1] used feature rectification during video target correction training. Hu et al. [2] proposed that combining multiple weak trackers may achieve better results than independent models. However, these methods still face challenges such as overfitting and excessive training time.

Despite significant progress in target tracking, cross-camera tracking remains a formidable challenge. Previously, it was viewed as a trajectory matching problem [3] limited by the bandwidth of centralized cloud servers, posing privacy and data security risks. Reliable blockchain-based cross-camera tracking frameworks are still scarce. To address these concerns, a blockchain-based online tracking framework for secure edge collaborative computing is proposed. Target tracking using heterogeneous nodes is a crucial research area within the Internet of Things. In practical applications, a single sensor may have performance defects, leading to incomplete performance. Moreover, the limited angle and viewing range of a single target tracker can also pose challenges. Li et al. [4] proposed a blockchain-enabled secure gateway architecture for internet communications security. Long [5] proposed a semi-supervised network for intrusion detection in the Industrial IoT. Cui et al. [6] proposed using combining the existing certificateless signcryption method with a fog architecture. This paper leverages target tracking from heterogeneous sources with multiple data processor co-scheduling.

In this paper, we propose a decentralized Ethereum blockchain technology-based solution for target tracking that utilizes feature location to correct deviation in position and establishes a communication system among agents in a peer-to-peer network using smart contracts. We also implement a trajectory optimization post-processing step to further enhance the accuracy of target location and reduce inter-frame jitter. Our approach is capable of functioning in scenarios where the target’s deformation and presence are unknown and leverages the organization of video target correction tasks through blockchain technology.

To sum up, the main contributions of our paper are as follows:

1. We propose a target tracking model for edge computing that is based on blockchain technology. This model takes advantage of diverse nodes and employs both software and hardware acceleration for feature extraction, as well as adaptive clustering, to achieve enhanced accuracy in tracking small objects.
2. A post-processing trajectory optimization is devised to address long-term occlusions, with the correction threshold set to allocate resources in an efficient manner to meet the demanding real-time requirements of complex environments.
3. Our model has been demonstrated to outperform existing models on the CarChase2 (TLP) dataset and BSA dataset.

The rest of this paper is organized as follows. The related work is reviewed in Section 2. The preliminary knowledge of our proposed model is listed in Section 3. The model proposed in this paper is discussed in detail in Section 4 and the experimental results are analyzed in Section 5. Finally, the whole work is summarized and future work is prospected in Section 6.

## 2. Related Work

This section briefly reviews cross-camera video analysis and the SIFT spatial information template, target location correction in video, and trajectory optimization post-processing.

### 2.1. Cross-Camera Video Analysis

Zhang et al. [7] studied the sharing of information between cameras. A mean-field game approach is proposed [8] to estimate the correlation of cross-camera video, but there are few studies of collaboration between cameras at distances. A cross-camera feature association module [9] for locating multiple human bodies was studied by Yang et al., but there was no further study of complex small targets.

A blockchain-based video analytics platform is a system that utilizes blockchain technology to provide a secure and decentralized platform for video analysis. In such a system, video data is recorded on a blockchain network, allowing for secure and transparent storage of the data. These data can then be analyzed using various video analytics algorithms. The results of these analyses can be stored on the blockchain as well, providing a tamper-proof and transparent record of the results. Hiding the real identity to protect the data security and privacy of IoT nodes is discussed by Liu et al. [10]. Liang et al. [11] proposed a spatial-temporal aware graph neural network for massive sensors. Liang et al. [12] proposed a framework for service recommendation in mobile edge computing environments. Liang et al. [13] proposed a privacy data protection and access control scheme, which boasts rapid response times and high efficiency in processing information. Han et al. [14] proposed to manage the access control policy for private data through the blockchain network and used a CP-ABE scheme to realize revocation and white-box traceability [15]. Li et al. [16] proposed to store video metadata as a blockchain transaction to support verification of video integrity and immutability. Sheng et al. [17] demonstrate collaboration between blockchain and video surveillance systems.

Much of the previous work has focused on recording the integrity of video, where blockchain is primarily used to verify video security or share video, while advanced video application research remains scarce.

### 2.2. Spatial Information Template

SIFT (scale-invariant feature transform) was originally proposed by Lowe et al. [18] for corner feature extraction tasks and has become a prevailing architecture in vision tasks. Many scholars have conducted research in relevant fields: Du [19] developed a scale-invariant PIIFD method based on pyramid matching to achieve better matching of different spatial features, Shen et al. [20] studied the global and local dependency modeling in Transformer structures, and Wang et al. [21] studied the spatio-temporal characteristics of data. Chen et al. studied the position information to enhance the visual representation [22], Cai et al. proposed a method to learn benign data distributions with polluted data [23], and Sarlin [24] developed a neural network to match spatial points, which could obtain highly accurate registration for the two groups of local features extracted.

**Figure 1:**
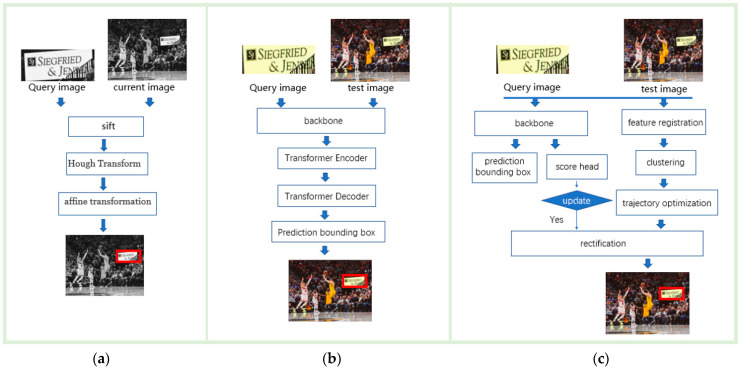
Three different target localization methods.

Our approach is influenced by SIFT, but has distinct differences. SIFT leverages the least-squares approach for affine transformation to determine the 3D rotation of a planar surface under orthographic projection. Conversely, our method employs a single adaptive k-means clustering to locate the target. As depicted in Figure 1, there are three methods for target localization: (a) a localization model utilizing local visual features, (b) a localization model utilizing both global and local visual features, and (c) the proposed tracking model.

### 2.3. Target Location Correction in Video

Correcting target location is a crucial aspect in the area of video target prediction. Recently, a new approach to tracking representation was introduced, known as the unified tracking graph. This representation combines both detections and tracks into a single graph, resulting in improved tracking performance. [25]. In addition, some recent trackers based on a graph neural network (GNN) [26] infer the relationship between nodes to filter out false detection. These methods are mostly dependent on the accuracy of location and target association. Another framework was proposed by Guo et al. [27], which used a multi-head attention to assign features of different representation spaces. Diao et al. [28] proposed a spatial-temporal attention graph convolution network (CRFAST-GCN) for traffic flow forecasting.

This paper presents an approach to video object tracking that combines target correction with a pre-trained tracker. The method utilizes information about the target’s location to enhance accuracy and stability, especially in situations where tracking score begins to drop. The proposed approach enhances its capacity to keep track of the target, even in challenging conditions such as occlusions and fast movements through the integration of target correction. Additionally, the utilization of a pre-trained tracker provides a strong foundation for the tracking process, enabling quicker and more effective tracking in real-time scenarios.

### 2.4. Trajectory Optimization Post-Processing

In video analysis, post-processing is an essential step for refining initial tracking results. Commonly employed post-processing techniques include trajectory prediction, scale or aspect-ratio penalty, and bounding box smoothing, among others, and they have been demonstrated to produce improved outcomes. The use of Kalman filter estimation as a post-processing step in video target tracking has been widely adopted [29]. Han et al. [30] introduced the motion-aware tracker (MAT) as a strategy to smoothly fill in tracking gaps caused by occlusions or blur. Dai [31] proposed to apply post-processing based on time series to post-processing of feature registration. Kurtz et al. [32] used a neural network to learn the trajectory and make predictions by combining off-line trajectory optimization with machine learning, enabling the redirection of the quadruped robot.

Most existing tracking models overlook target relocation. This work aims to address this issue by predicting a single bounding box per frame and optimizing the target trajectory to align with groundtruth.

## 3. Preliminaries

In this section, we cover the following topics: blockchain and the smart contact, the corner feature template, acceleration of the feature extraction process, localization through clustering, and trajectory optimization.

### 3.1. Blockchain and Smart Contract

Blockchain is a decentralized and distributed digital ledger that records transactions on multiple computers in a secure and transparent way [33,34]. Each block contains the transaction content and timestamp and references the hash of its previous block [35].

A smart contract is a self-executing contract with the terms of the agreement between buyer and seller being directly written into lines of code. These contracts run on a blockchain network and are stored on multiple computers, making them tamper-proof and transparent. They allow for automatic execution of the terms of the agreement without the need for intermediaries.

### 3.2. Feature Template

**Figure 2:**
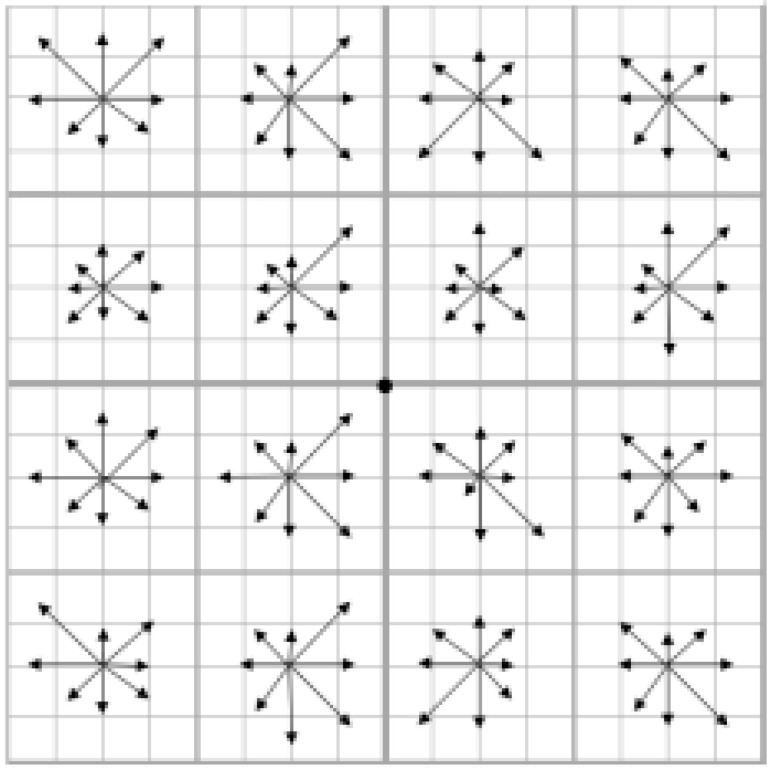
SIFT local feature template.

The basic concept behind the feature template is to use the extreme points as potential corners in a multi-scale pyramid, meaning that the target image’s contour position is selected as the feature point and the information in both scale and direction is taken as the feature vector for these potential points. The SIFT feature template is preferred by scholars because it is scale-invariant and provides more effective information for object matching with a high number of feature points and strong robustness. However, the drawback is its complex calculation, which slows down processing speed. Figure 2 displays the gradient feature information of one key point, where the information on a key pixel represents 16 grids and 8 gradients in the surrounding area.

Multi-core hardware implementation algorithms can provide better performance than CPU alone. Three open-source datasets: cudasift, gpusift [36], and popsift [37], were studied in a multi-core environment, with cudasift being the fastest and gpusift following; however, they could not extract scale-invariant features effectively. Popsift had better feature extraction capabilities, making it the choice for the hardware acceleration module to solve the speed issue from heavy calculations. The host copies input to device memory, calls the feature extraction function, and then copies the output to system memory.

In addition, existing code can be accelerated using multi-process or OpenMP and hardware parallel computing. The communication between the device and host is slow, so a GPU–CPU interaction-based application is needed to reduce overhead. This paper uses a synchronous multi-process method for data parallelism on multiple GPUs in the parallel experimental environment. 

### 3.3. K-Means Clustering

K-means clustering is a clustering algorithm that originally comes from signal processing and aims to divide n observations into k (k ≤ n) sets, s = ($s_{1}{,s}_{2},\ldots {,s}_{k}$), thus minimizing the sum of squares in the cluster. The data points in the cluster are considered to be more similar to each other than those belonging to other clusters. Given a group of observations ($x_{1}{,x}_{2},\ldots {,x}_{n}$), the end condition of iteration is shown in Formula (1).

(1) $$\arg\underset{s}{\min}\overset{k}{\underset{i=1}{\sum }}\underset{x\in s_{i}}{\sum }{\left\|x\;-\;\mu_{i}\right\|}^{2}=\arg\underset{s}{\min}\left|s_{i}\right|{\mathrm{Var}\;s}_{i}$$

where $\mu_{i}$ is the mean value of point $s_{i}$ and ${\mathrm{Var}\;(s}_{i})$ is the variance of point $s_{i}$ and is equivalent to minimizing the paired square deviation of points in the same cluster. Because the total variance is constant, this is equivalent to maximizing the sum of squares of the deviations between the midpoints of different clusters.

### 3.4. Kalman Filtering

**Figure 3:**
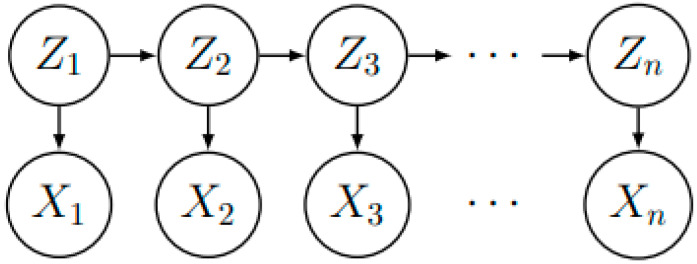
A discrete hidden Markov model.

Rudolf E. Kalman first introduced Kalman filtering, which aims to minimize errors to enhance measurement accuracy. The method controls inputs and a series of sensor measurements to improve the estimate of the system state. This state-space approach also makes it easier to implement the filter in a discrete format, contributing to its widespread use. The Kalman filter is like a discrete hidden Markov model (HMM), where the observed sequence ($x_{1}{,x}_{2},\ldots {,x}_{n}$) is combined with a series of hidden states ($z_{1}{,z}_{2},\ldots {,z}_{n}$), as shown in Figure 3.

Different from discrete state HMM, each hidden state of the Kalman filter is modeled as a continuous random variable with multivariate normal distribution. Define $p\left(x_{1:n}{,z}_{1:n}\right)=p\left(x_{1}|z_{1}\right)\prod_{j=2}^{n}p\left(z_{j}|z_{j-1}\right)p\left(x_{j}|z_{j}\right)$, where ${p(z}_{1})$ is the initial distribution, $p\left(z_{j}{|z}_{j-1}\right)\;\mathrm{is}\;$the transfer distribution, also known as the process model, and $p\left(x_{j}|z_{j}\right)$ is the measurement model.

The Kalman filter assumes that the calculated error follows the normal distribution, and the greater the width (variance) of the distribution, the higher the uncertainty of the prediction value. It can be used as a smoother, filter, or predictor for a wide range of tracking and navigation systems.

## 4. Target Tracking System Model

The correction procedure for visual tracking is separated into two parts: feature-based target location and tracking and time-series based target correction. This proposed method incorporates the benefits of blockchain technology and spatio-temporal information for visual tracking. This section outlines the function of the blockchain system, outlines the positioning model, explains the clustering algorithm and the trajection optimization, and finally introduces the proposed single target tracking and correction model.

The system utilizes smart contract to manage the recording and retrieval of target tracking data. The smart contracts will enforce the rules for recording data and ensure that all nodes have an accurate and up-to-date copy of the blockchain database. The target tracking data stored on the blockchain network will be used to visualize the movements of the targets in real-time. This can be done using a web interface or a dedicated application. The visualization will allow users to monitor the location of the targets in real-time and see their movements over time.

### 4.1. The Blockchain

The blockchain system plays a crucial role in ensuring the security and transparency of the target correction process. A system where personal identity information is stored in a decentralized, secure, and tamper-proof manner, making it possible to track and verify the identity of individuals involved in target tracking. Smart contract is a self-executing contract that automatically enforce the terms of an agreement, which can be used to track the fulfillment of specific targets or goals. Smart contracts can be programmed to automatically enforce specific conditions and actions related to target tracking. For example, in logistics and transportation, a smart contract can be set up to automatically release payment to a delivery company when a delivery target, such as the arrival of a package at a specific location, is confirmed through automatic visual target tracking.

In this paper, the miner with the highest cluster score will have the opportunity to correct the target and record the updated information on the blockchain. Machine learning algorithms and predictive analytics can be used to analyze data on the blockchain and provide insights into potential target attainment, helping organizations to optimize their target tracking efforts.

**Figure 4:**
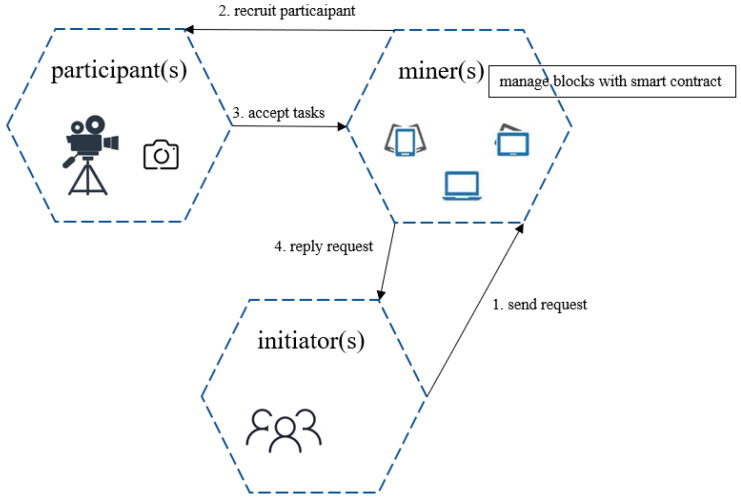
Roles of target tracking system.

The architecture of the blockchain-based target tracking system has three roles: miner, initiator, and participant, as Figure 4 shows. In the blockchain-based target tracking system, participants provide images of the search area from different angles to assist miners in finding target features and transmitting correction information to initiators.

Miners act as intermediaries between initiators and participants, matching requests with participants and maintaining stable operation in the blockchain system. Miners can be servers from various public third-party platforms. The initiator is the user who initiates the target tracking task. They send the task to the miners. The miners then write the smart contract and transaction data onto the blockchain. The tracking results are directly returned by the participant to the initiator, who then uses the target coordinates obtained from the blockchain. A participant is a camera that can assess miner recruitment and accept tasks. It retrieves feature location task parameters and smart contract information from the blockchain.

Blockchain technology enhances the security of visual tracking by providing an immutable record of target movements and data. Its decentralized structure eliminates the risk of a single point of failure, making the system more secure. Real-time access to visual tracking data is facilitated, enabling stakeholders to easily monitor and verify their targets. The integration of blockchain and spatio-temporal information improves the accuracy and timeliness of target records, especially in complex and dynamic environments where accuracy is crucial.

#### The Smart Contract

For the real-time tracking task, a smart contract to manage the recording and retrieval of target tracking data could be designed to use features instead of image data. This would help to address the restriction on image data storage and transmission. Each node in the network would extract relevant features from the image data it collects and record these features in a smart contract. The features could include, for example, the target’s location, size, and shape. The smart contract would validate the features recorded by the nodes to ensure that they meet certain predefined criteria. For example, a smart contract could validate that the features are accurate and consistent with the target tracking task. The validated features would then be stored in the smart contract, replacing the need to store the image data. Storing features instead of image data would significantly reduce the storage space required and also speed up the data retrieval process.

The purpose of this system is to demonstrate the potential benefits of utilizing blockchain technology in target tracking applications. The system utilizes real-time sensor data collected from cameras attached to the targets. The cameras act as the primary data collection sources, capturing real-time information about the movements of the targets. The data is transmitted to a central server through a secure wireless communication protocol, such as WiFi or a cellular network. The central server serves as the repository for the target tracking data, ensuring that only authorized users have access to it. The target tracking data is recorded in a blockchain database, which is maintained by a decentralized network of nodes. These nodes use consensus algorithms to ensure the integrity and security of the blockchain database. The use of a blockchain network provides a secure and tamper-proof record of the target’s movements, ensuring that the data cannot be altered or deleted without consensus from the network.

### 4.2. Positioning Model Based on Feature Registration

**Figure 5:**
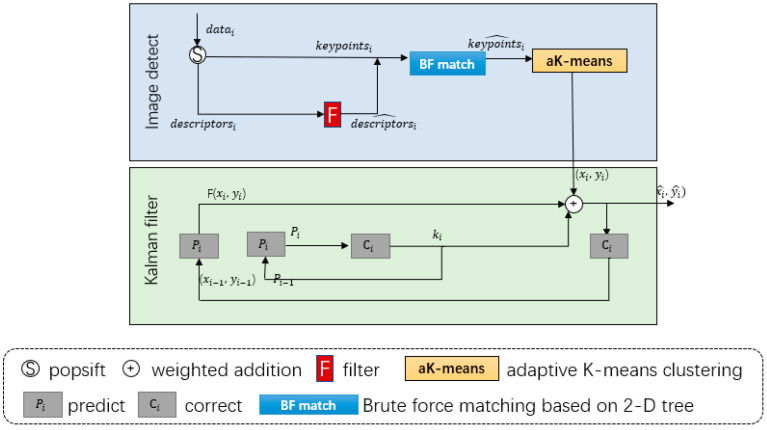
Logic diagram of video object location model.

This paper presents a target location model (shown in Figure 5) that utilizes feature registration. The model extracts a group of features from the input image and performs keypoint extraction and feature vector calculation across various scales. A filtering algorithm is applied to identify target features by comparing the nearest and next nearest neighbor distances. The target template keypoints and image frame keypoints are matched to register the features, with the cluster center having the highest matching degree serving as the detection result. The trajectory of the cluster center is optimized for precise tracking.

Additionally, the paper employs a dynamic adaptive k-means clustering algorithm to group similar targets based on their movement patterns. The number of cluster centers is calculated through Formula 2, and the clustering model is optimized through appropriate hyper-parameter tuning, resulting in a fully automated positioning model capable of locating the target.

(2) $$W=\min\left(\lceil \frac{S}{T}\rceil ,\;T-1,\;K+1\right)$$

The steps to build the clustering model are in Algorithm 1, leading to the creation of a fully automated positioning model that can locate the target.
**Algorithm 1:** Adaptive k-means clustering.
**Input**: P denotes the features extracted from the search region after filtering; S denotes the number of all features extracted from search region; K is the hyperparameters of upper limit;
**Output**: cluster center which has the most points1:N ← numbers of points 2:W ← min{K, T-1, ⌈S/T⌉}.3:**initialize** centers [W]4:S = Null5:**Repeat until** centroids C =$\;\left\{c_{1}{,c}_{2},\ldots {,c}_{k}\right\}$ is not changed6:  **for** i = 1 **to** N **do**7:    **for** j = 1 **to** W **do:**8:     dis = ComputeDist($P_{i},\mathrm{centers}\;[W]$)9:      **if** dis < minDis **then**10:        minDis = dis11:        index = i12:    $S_{\mathrm{index}}\cup {\;P}_{i}$, assign $P_{i}$ to its cluster set $S_{\mathrm{index}}$
13:  **end for**
14:  num [W] ← record the sum if points in the same cluster15:  divide the entries of centers by num [W] to get the new center’s coordinates16:**until** the distances of all clusters do not change17:score ← ratio between within-cluster dispersion and between-cluster dispersion18:center = 019:**for** j = 1 **to** W **do:**20:  **if** center < centers [j] **then**21:    center = centers [j]22:**return** center’s coordinates, score

### 4.3. A Trajectory Optimization Model

In cases where the target is in a continuously observed time series, its position can be estimated based on its location in previous frames. However, this method has two drawbacks. First, it can be challenging to determine the target’s motion direction and speed in various circumstances, leading to difficulties in establishing a proper motion model. Second, the presence of noise in the data can hinder picture clarity and affect the accuracy of target localization. To address these issues, we incorporate a Kalman filter as a post-processing model. Table 1 displays a nomenclature table containing a list of variables and their corresponding meanings.

The noise in images can impact the accuracy of the model. Two similar images show that the two cluster centers wander around the target, causing jitter in the bounding box across frames. To improve accuracy, a trajectory optimization model is used, which predicts current target position based on previous frames and corrects current measurement. The model uses a weight to minimize error variance.

The Kalman filtering mechanism is divided into prediction and correction stages. During prediction, the target position and speed are calculated, assuming uniform speed and normal distribution measurement error. The resulting variables, $z_{1}$ and $z_{2}$, represent target position and speed, respectively, as in Formula 3.

(3) $$z_{2}=\dot{z_{1}}=\frac{{z_{1}}_{k+1}\;-\;{z_{1}}_{k}}{\Delta t}$$

The prediction phase includes the noise in the prediction data. The noise, represented by v, covers various sources such as random movement of the lens and image sensor acquisition processes. It is expected to have a normal distribution with a mean of zero, as shown in Formula 4.

(4) $$v\;\sim \;N\;\left(0,\;\sigma^{2}\right){,\sigma }^{2}=E\left[\begin{array}{cc} v & v^{T} \end{array}\right]$$

where p(v) represents the probability of the noise value and $\sigma^{2}$ is the variance. The model iteratively predicts the noise variance in the measurement time series. The smaller the variance, the less uncertainty in the prediction, leading to a more accurate feature location result. The prior noise in the prediction data is calculated using Formula 5.

(5) $$v_{k}{}^{-}{=v}_{k-1}{\;+\;\sigma }^{2}\;$$

The predicted value, $Y_{k}$, is calculated as Formula 6.

(6) $$Y_{k}{=A\;\times \;Z}_{k}{\;+\;v}_{k}{}^{-},{\;Z}_{k}={\left[\begin{array}{cc} z_{1} & \dot{z_{1}} \end{array}\right]}_{k}$$

where A is the state matrix of the target moving at a uniform speed that can be calculated as $\left[\begin{array}{cc} 1 & 1\\ 0 & 1 \end{array}\right]$.

Step 3: After obtaining the prediction, ${\hat{Y}}^{-}$, and errors, $v_{k}{}^{-}$, the current measurement results are updated by balancing the weight of the prediction and the measurement based on the prediction error to minimize it. The weight, $k_{k}$, can be calculated as Formula 7.

(7) $$k_{k}=\frac{v_{k}{}^{-}}{v_{k}{}^{-\;}{+r}^{2}}$$

After the calculation of the Kalman coefficient is completed, we can update the measurement as a weighted sum of current location value and previous measurement, that is, the updated measurement, ${\hat{Y}}_{k}$, can be calculated as Formula 8, and $X_{\mathrm{MEA}}$ is the result of target location model.

When the Kalman coefficient calculation is finished, the measurement can be updated as a combination of current location and previous measurement with a weight. The updated measurement, ${\hat{Y}}_{k}$, can be calculated using Formula 8, and $X_{\mathrm{MEA}}$ is the target location result.

(8) $${\hat{Y}}_{k}{=\hat{Y}}_{k-1}{+k}_{k}\left(X_{\mathrm{MEA}\;}-{\hat{Y}}_{k-1}\right)$$

The prediction error, $v_{k}$, should be updated for the next prediction, which can be performed using Formula 9.

(9) $$v_{k}=\left({I\;-\;k}_{k}\right){\;v}_{k}{}^{-}$$

The trajectory optimization model uses $D_{k}$ (distance between target location and current measurement position) to calculate the ideal speed of the target. If $D_{k}$ exceeds the ideal speed, the approximate prediction method is used for correction. If $D_{k}$ is greater than the threshold, steps 2 and 3 are repeated until $d_{k}$ is less than the threshold, γ; finally, the updated measurement is obtained. The number of cycles, i, is shown in Formula 10.

(10) $$d_{k}{=D}_{k}\;-\;\gamma \;\times \;i$$

The proposed trajectory optimization is effective for when the target is temporarily unseen due to occlusion, movement, etc., and reappears elsewhere. To avoid false detections affecting the optimization, conditions are added. The detailed steps are outlined in Algorithm 2. The “×” represents a matrix multiplication.
**Algorithm 2:** Trajectory Optimization based on the Kalman filter.
**Input**: data denote the result of detection process to be processed; γ is the threshold of distance;
**Output:** points denote the center of the target1:Initial first distance as zero 2:Initial $Z_{k}\;\leftarrow \left[\begin{array}{cc} z_{1} & \dot{z_{1}} \end{array}\right]$
3:**for** $X_{\mathrm{MEA}}$ **in** dataset **do**4.  $Y_{k}{=A\;\times \;Z}_{k}{+v}_{k}{}^{-}$
5:  $v_{k}{}^{-}\;\leftarrow {\;v}_{k-1}{+\sigma }^{2}$
6: **if** distance ≤ γ **then**7:  $k_{k}\;\leftarrow {\;v}_{k}{}^{-}{\backslash (v}_{k}{}^{-}{+r}^{2})$.8:  ${\hat{Y}}_{k}\;\leftarrow {\hat{Y}}_{k-1\;}{+k}_{k}{(X}_{\mathrm{MEA}\;}{-\hat{Y}}_{k-1})$.9:  $v_{k}\;\leftarrow \;\left({I\;-\;k}_{k}\right){\;v}_{k}{}^{-}$.10:  **else if** distance > γ **then**11:    **repeat**
12:      $Y_{k}\;\leftarrow {\;A\times Z}_{k}{+v}_{k}{}^{-}$
13:      $v_{d}{}^{-}\;\leftarrow v_{d-1}{+\sigma }^{2}$
14:      $k_{k\;}\leftarrow v_{k}{}^{-}{\backslash (v}_{k}{}^{-}{+r}^{2})$
15:      $v_{k}\;\leftarrow \left({I\;-\;k}_{k}\right){\;v}_{k}{}^{-}$.16:      distance ← distance -γ17:    **until** distance ≤ γ 18:    ${\hat{Y}}_{k}\;\leftarrow {\hat{Y}}_{k-1\;}{+k}_{k}{(X}_{\mathrm{MEA}}{\;-\hat{Y}}_{k-1})$
19:  **return** ${\hat{Y}}_{k}$

### 4.4. A Single Target Tracking Correction Model

The proposed model in this paper combines the positioning model, clustering algorithm, and trajectory optimization to form a solution for single target tracking and correction. The use of blockchain technology and the spatio-temporal information enhances security, transparency, and accuracy in target correction and tracking.

**Figure 6:**
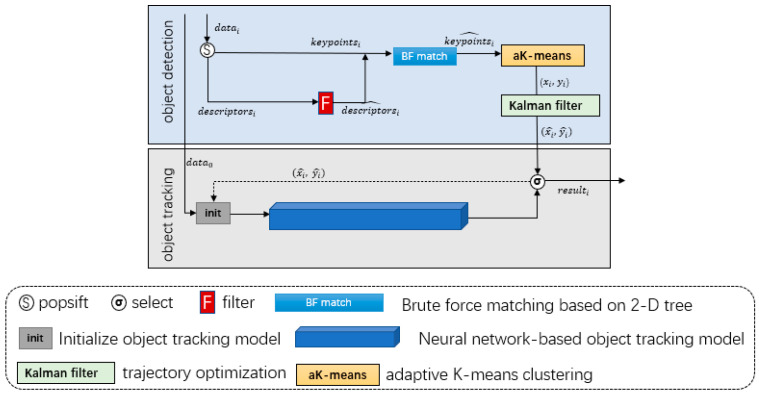
Flow chart of model based on feature registration correction.

The results of experiments with two state-of-the-art tracking models show that the tracking model performs well before occlusion, but correction is needed when confidence is reduced. The correction model is based on feature registration, as shown in Figure 6. Success is the number of successful detections and “λ” is the correction threshold. The model starts detecting the target when the confidence of the tracking model results is below 0.3. If the detection result falls within the bounding box of the tracking model, it means that the tracked target has the characteristics of the detected target, and the results of the tracker can be trusted. To avoid false detections, the tracking model is initialized with the tracking box of high confidence from the last tracking model and the current detection result when the distance between successive detections is less than γ for λ times. The detailed steps to establish the single target tracking deviation correction model are shown in Algorithm 3.
**Algorithm 3:** Video Target Tracking and Correction.
**Input:** $\left(\hat{x_{i}},\;\hat{y_{i}}\right)$ denotes the result of detection model after trajectory optimization; λ is the hyperparameter of detection success threshold; γ is the hyperparameter of distance threshold;
**Output:** result bounding box1:Initial Tracking model 2:success = 03:**for** frame **in** dataset **do**4:  BBox, conf_score ← t rack (a frame)5:  **if** conf_score ≥ 0.99 **then**6:   pre_h, pre_w, result ← Bbox7:  **if** conf_score ≤ 0.3 and point $\left(\hat{x_{i}},\;\hat{y_{i}}\right)$ doesn’t fall in the BBox **then**8:  $\left(\hat{x_{I}},\;\hat{y_{i}}\right)\;\leftarrow$ detect (a frame)9:  **if** the distance between point$\;\left(\hat{x_{I}},\;\hat{y_{i}}\right)$ and point $\left(\hat{x_{i-1}},\;\hat{y_{i-1}}\right)$ **is less than** γ  **then**10:     success = success + 111:    **else:**
12:     success = 013:    **if** success > λ  **then**14:     Initialize Tracking model with the detected point $\left(\hat{x_{I}},\;\hat{y_{i}}\right)$ and pre_h, pre_w15:     result ← I, $\left(\hat{x_{i}},\;\hat{y_{i}}\right)$, pre_h, pre_w16:  **else:**
17:    result ← BBox18:  **return** result

## 5. Experiment

### 5.1. Data Set and Evaluation Indicators

#### 5.1.1. Datasets

The proposed models are evaluated using both manually labeled and public datasets. The manual annotation dataset, referred to as BSA, consists of three segments from sports events on YouTube. The targets in this dataset are ads on basketball stands, with dimensions of 1080 × 1920 pixels, a frame rate of 30 fps, and a duration of approximately 3 min. The video data is annotated every 10 frames through the EasyData platform, resulting in a validation dataset of 1992 pieces. To showcase the practicality of the model, segments are selected based on specific requirements, and three basketball game videos with complex backgrounds from the internet are used as the experimental subjects, particularly video clips featuring repeated object appearance and disappearance.

**Figure 7:**
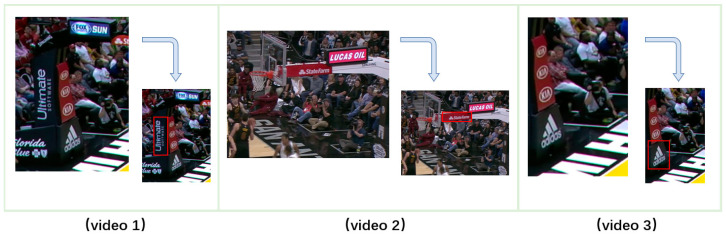
Three different targets in the BSA dataset.

As for the TLP [38] dataset, it is a long-term visual tracking database with video clips averaging 8 min in length. The performance of currently popular trackers is influenced by both the difficulties in video sequences and video length. In comparison to short-term tracking tasks such as TrackingNet [39], TLP offers longer and more practical continuous annotation sequences. Therefore, this study primarily conducts experiments using TLP for more reliable and complete results. The target selections in the videos are as follows: In video 1, the black "ultimate software" advertisement on the basketball post is chosen, in video 2, the red "statefarm" advertisement behind the basketball rim is selected, and in video 3, the black "adidas" advertisement near the ground at the front of the basketball stand is identified as the target. These selections are illustrated in Figure 7.

#### 5.1.2. Experimental Environment

This paper tests the model in three experimental setups, as outlined in Table 2. The experiment for the single video detection model is conducted using an NVIDIA GeForce RTX 3050 graphics card, while multiple multi-core NVIDIA Telsa graphics card environments are utilized for the multi-process parallel experiment. The parallel experimental environments are shown in Table 2.

#### 5.1.3. Evaluation Criteria

In this paper, accuracy, recall, and F1 score are expressed as evaluation criteria to evaluate the proposed model. Their definitions are shown in Formulas 11–14.

(11) $$\mathrm{Precision}=\frac{\mathrm{TP}}{\mathrm{TP}+\mathrm{FP}}$$

(12) $$\mathrm{Recall}=\frac{\mathrm{TP}}{\mathrm{TP}+\mathrm{FN}}$$

(13) $$F1\;\mathrm{score}=2\;\times \frac{\mathrm{recall}\;\times \mathrm{precision}}{\mathrm{recall}+\mathrm{precision}}$$

(14) $$\mathrm{uccess}=\frac{\left|\mathrm{bounding}\;\mathrm{box}\cap \mathrm{gt}\right|}{\left|\mathrm{bounding}\;\mathrm{box}\cup \mathrm{gt}\right|}$$

The precision is the ratio of correctly detected targets to the total number of detected targets, while recall is the ratio between the number of correctly detected targets and the total number of targets in the dataset. The F1 score is the reciprocal average of precision and recall. One-pass evaluation (OPE) is a technique used to estimate the performance of an algorithm; it can be defined as a method for assessing the performance of a model by using a single evaluation metric, such as success or precision, based on a single pass through the data. Success refers to the overlap score of the bounding box in tracking objects in video sequences. In this article, the term “robustness” specifically refers to the ability of the model to maintain its performance and provide accurate results in the presence of various types of noise or distortions in the data in an open-set scenario. The evaluation tool provided in [39] is used to compute the score of each model.

#### 5.1.4. Baseline

We present two baseline tracking models: Siamese RPN++ [40] and STARK [41]. Siamese RPN++ is a semi-automated tracking network, while STARK is an automated target retrieval tracking network. Both models were evaluated on road scene and sports event datasets. In Section 5.3, we compare the proposed detection models.

SiamRPN++’s key accomplishment is using a sampling strategy where the search area is centered around the target’s location in the previous frame, and then cross-correlating the target template and search area using convolutional features to track the target. Meanwhile, STARK introduces an efficient tracking algorithm for the best performance. It first mines the data’s spatial-temporal information using a transformer to create a dynamic template of changes, then calculates the heatmap of the tracking target in the current search area.

To ensure fair comparison performance among all the comparison methods, the experiment’s super parameters were carefully tuned for each method according to the relevant references.

### 5.2. Comparison with Traditional Methods

**Figure 8:**
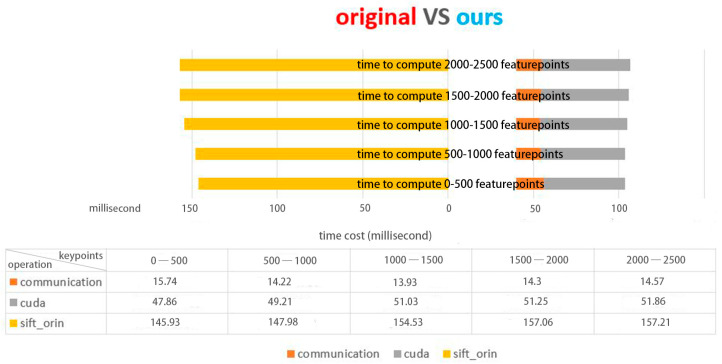
Time consumption on feature extraction.

**Figure 9:**
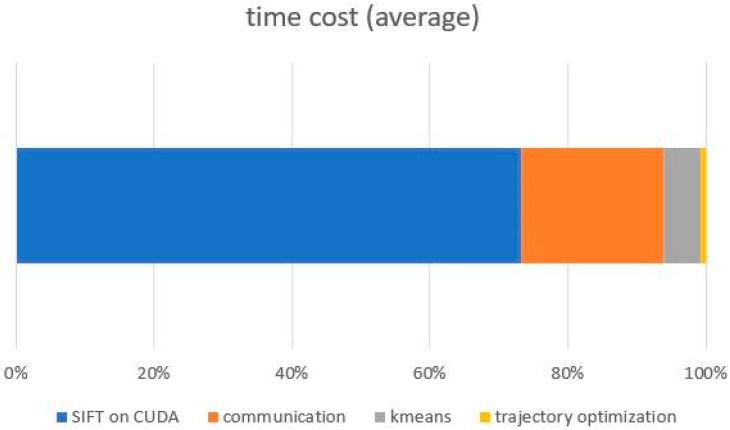
Time Cost of each stage (ours).

**Figure 10:**
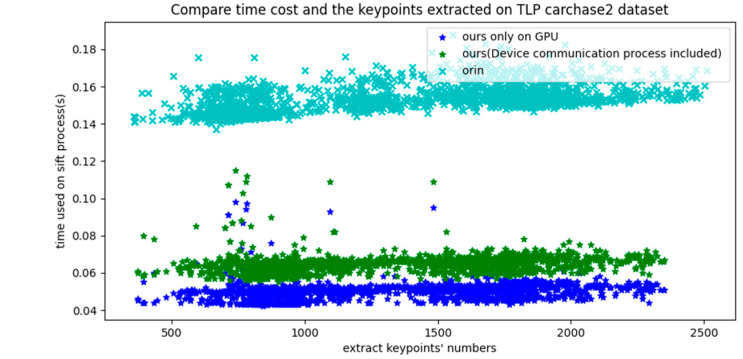
Time consumption on feature extraction in detail.

The RANSAC algorithm is a mature and effective target detection model based on feature registration. However, experiments show that this method has difficulty reliably identifying small, fuzzy moving targets. A horizontal comparison was made between the original model implementation and the proposed model on the open real-world TLP dataset. Figure 8 displays the distribution of the number of feature points in the CarChase2 (TLP) dataset and the time cost of the corresponding feature extraction in experimental environment 1. As evident from Figure 8, Figure 9 and Figure 10, the computing time cost of our model ranges from 1000 to 3000 feature points, with the time for communication transmission between the device and host taking up roughly 20 milliseconds, which is approximately one-third of the total time cost. Our implementation exhibits a three-fold improvement in speed compared to the original method. The proposed model performs significantly better in terms of time compared to the original method. Figure 10 provides a detailed analysis of the time cost in the main stages of the proposed model.

In experimental environments 2 and 3, data parallelism is achieved using the multi-process method based on GPUs. The current frame is assigned to idle devices for feature template extraction based on its current frame number, and the detection results are calculated in the multi-core CPU after the results are transmitted to the host computer. However, the output bounding box computed by this method has strong jitter, so a trajectory optimization model was added. A Kalman filter calculation is then performed after synchronizing the results.

The design of the model, with its simple weak coupling, results in time bottlenecks only during the synchronization of each result transmission. The experiment shows that every time a GPU device is added to a single-core CPU host, the computing speed of a single model decreases by 40%, while the total computing speed increases by 0.7 times. However, in a multi-core environment, acceleration restrictions are reduced. When using eight GPU devices, the computing speed of a single model decreases by only 10%, and the total computing speed increases by about 0.9 × N (where N is the number of devices). Table 3 shows that the performance of our method is better than that of traditional feature-based registration algorithms.

**Figure 11:**
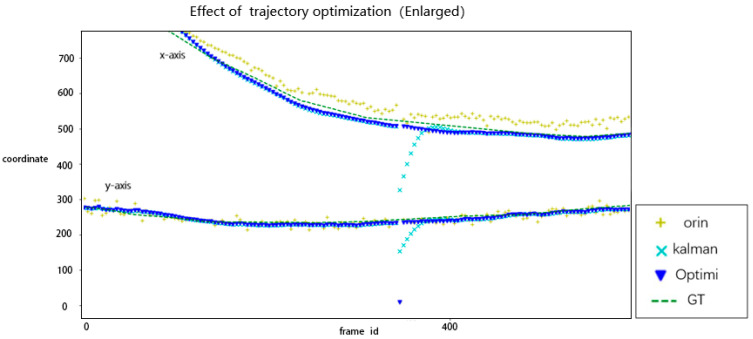
Locations optimized by trajectory optimization (partial).

**Figure 12:**
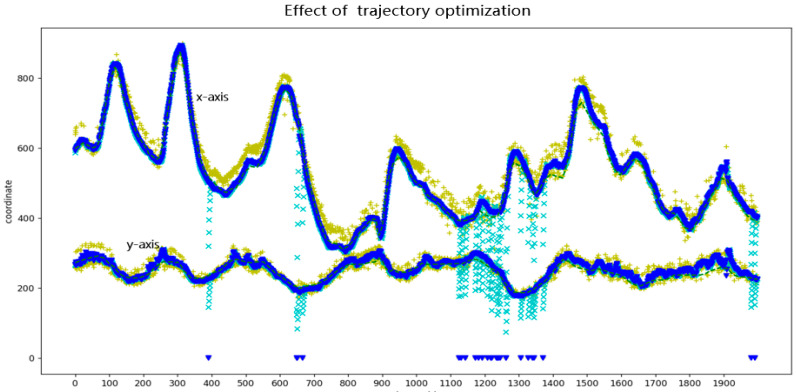
Locations optimized by trajectory optimization.

The location results of CarChase2 after optimization using the trajectory optimization model are displayed in Figure 11 and Figure 12. The graph compares the “orin” (time series from the target location model), “kalman” (barycentric coordinate of the target after two-dimensional Kalman filtering without iteration), “Optimi” (barycentric coordinate of the trajectory optimization model), and “GT” (annotation value).

### 5.3. Combination with Existing Technology

**Figure 13:**
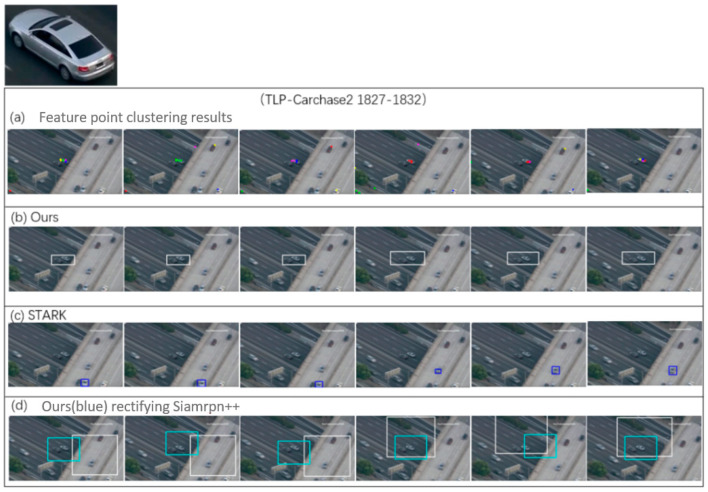
Performances of different models.

**Figure 14:**
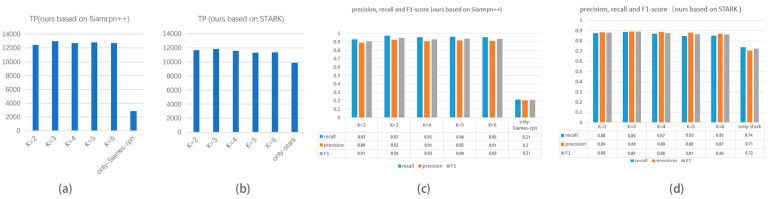
Test results of models under different K values.

The temporal robustness evaluation (TRE) metric is a suitable metric for evaluating tracking algorithms that aim to maintain a consistent and accurate estimate of the object’s location. However, the one-pass evaluation (OPE) is useful in cases where the target may disappear and reappear in the image sequence, as it provides a global evaluation of the algorithm’s performance across all frames. Our approach uses the bounding box in the first frame of the dataset as the only template to maintain a consistent and accurate estimate of the object’s location. The effectiveness of our proposed model is demonstrated on the TLP dataset. As the video duration increases, the model shows greater robustness compared to single object tracking models, which lose track of targets. To compare performance, we present the accuracy and recall of the CarChase2 (TLP) dataset using various methods in Figure 13. The internal parameter, K, was selected based on the results in Figure 14.

To enhance the performance of our model, we examine the impact of various parameters on its performance across two datasets, including both manually annotated datasets and CarChase2. One of the parameters we focus on is the super-parameter K, which is a parameter that appears in Algorithm 1 and represents the maximum number of objects that can be grouped. We compare the results of the Fl-score metric for different values of K ranging from 2 to 6. The results are displayed in Figure 14.

Figure 14 shows that our proposed scheme surpasses other baseline methods in terms of precision and recall. Compared to STARK, our scheme has a higher accuracy and recall, by 18.1% and 10.8%. Our model’s performance is measured against the general Siames-rpn model on the CarChase2 dataset, recording 68.42% accuracy and 71.75% recall, which are lower compared to our scheme. Our scheme outperforms single neural network models by using scale-invariant features for improved performance. In case of occlusion or disappearance of the target, feature registration is employed for recapture, resulting in a more reliable tracking performance. In the test dataset, there were three similar silver-white cars to the target, and as seen in Figure 12 (right), the adaptive tracking model performed best with K = 3 (1%+).

**Figure 15:**
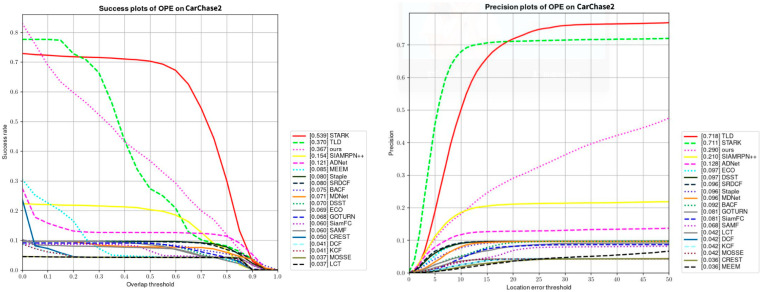
Comparison with other methods.

As demonstrated in Figure 15, the performance of our proposed model on the CarChase2 dataset is compared with that of other models; 70% of the frames in our proposed model have an overlap score greater than 0.1. The performance of our proposed model was also verified on the BSA dataset. When the clustering center of the model was not found in the detection result box of the neural network, the detection result of feature clustering was used as the center of gravity of the detection box, and the target was relocked by combining the weight of the previous location with high confidence and the initial target. Table 4 verifies the robustness of our model. To ensure fair performance comparison, the average of the results from multiple experiments on different targets was taken for all video clips.

## 6. Discussion

In summary, we have created a target tracking system using the tracking-by-detection theory in a blockchain-based photography system and calculated the weighted target location for trajectory prediction. The scale-invariant features provided by each miner are used to detect the target’s visibility and speed through a smart contract, which can also be utilized for visual monitoring.

Moreover, numerous decentralized robot applications can be studied in the context of blockchain-based analysis tasks, such as the synchronization, dispersion, and aggregation of 3D behaviors and various coordination methods for robotics.

Furthermore, multi-angle feature fusion is a promising area of research as two participants form an angular gap, requiring the integration of multi-sensor data. The blockchain infrastructure supporting multiple sensor communication holds great potential in providing the best answer. Our experiments, outlined in Section 4, demonstrate this advantage by introducing an additional independent trajectory optimization and only considering results from miners with multiple target hits in a short time.

Our research brings together tracking correction, trajectory optimization, and blockchain, opening up new avenues for exploring the collaborative communication capabilities of computer vision systems using multiple sensors. It is important to note that the integration of blockchain and target tracking is a new and developing field, facing technical difficulties such as real-time processing for target tracking in decentralized systems and privacy concerns for sensitive tracking data on public blockchains. Nevertheless, the combination holds great potential for various applications and is expected to see ongoing research and development in the future.

## 7. Conclusions

The integration of blockchain technology and visual tracking has the potential to revolutionize the way targets are monitored and tracked in real-world situations. In this study, the input sensor data is processed by miners based on the tracking-by-detection theory to provide efficient target location in images. Adaptive clustering is used to analyze data and determine the target’s location in situations where historical information is lacking, while trajectory optimization and post-processing address inter-frame jitter. The accuracy of target correction is crucial for effective tracking, as traditional tracking methods can be affected by occlusion and blur, particularly for fast-moving targets. To overcome these limitations, decentralized data processing and storage can be used. By incorporating consensus algorithms in the tracking process, real-time correction and adjustment is made possible, meeting industry requirements. Through experimental analysis using various datasets, the proposed algorithm has shown improved recall and precision compared to existing tracking models. Experimental analysis was carried out for various datasets; the location model proposed resulted in recall of 51% (27.96+) and precision of 66.5%( 40.04+) in the CarChase2 dataset and recall of 85.52 (11.75+)% and precision of 47.48 (39.2+)% in the BSA dataset. Moreover, the proposed video target tracking and correction model performs better than the existing tracking model, showing a recall of 97.1% and a precision of 92.6% in the CarChase2 dataset, and an average recall of 75.9% and mAP of 82.87% in the BSA dataset, respectively. However, the algorithm still struggles with processing blurred images, and future research will concentrate on enhancing feature recognition. Furthermore, the blockchain technology developed has the potential to be extended to a wider range of data types, including audio and character data, in addition to image processing.

## Figures and Tables

**Table 1:** Nomenclature.

Variables	Meaning
$z_{1}$	target location
$z_{2}$	target speed
Δt	amount of change in time
v	locate noise
σ	standard deviation
$N\;\left(0,\;\sigma^{2}\right)$	normal distribution
$v^{-}$	prior noise
Y	predicted target location
A	state matrix
$k_{k}$	Kalman gain
$X_{\mathrm{MEA}}$	target location to be corrected
D	distance between Y and $X_{\mathrm{MEA}}$
γ	target’s ideal moving speed
i	Iteration number
d	distance between $D_{k}\;\mathrm{and}\;\gamma \times i$

**Table 2:** Details of Experimental Environment.

Experiment Environment	Processor	Graphics Card
environment 1	AMD Ryzen 7 5800H 3.20 GHz	AMD Radeon(TM) Graphics + NVIDIA GeForce RTX 3050 Laptop GPU
parallel environment 2	Intel Xeon Platinum 8259CL 2.50 GHz	NVIDIA Telsa T4 × 8
parallel environment 3	Intel Xeon Platinum 8259CL 2.50 GHz	NVIDIA Telsa T4 × 4

**Table 3:** Comparison above CarChase2 dataset.

Model	Recall (%)	Precision (%)
orin	23.04	26.51
ours	51	66.55

**Table 4:** Experimental comparison on the BSA dataset.

BSA Dataset	Video 1		Video 2		Video 3	
Number of Frames	8550		6300		5070	
Model	Recall (%)	Precision (%)	Recall (%)	Precision (%)	Recall (%)	Precision (%)
SIFT + RANSAC Feature Location(ours)	44.7	9.6	96.3	8.9	94.8	4.5
	83	49	95	50	78	41
STARK Tracking (ours)	35	90	70	90	70	90
	98.7	46.9	91.3	89	91.9	58.6

## Data Availability

The original data can be obtained from the open access online dataset by Moudgil et al. [38].
